# Genetic architecture of white striping in turkeys (*Meleagris gallopavo*)

**DOI:** 10.1038/s41598-024-59309-8

**Published:** 2024-04-18

**Authors:** Ryley J. Vanderhout, Emhimad A. Abdalla, Emily M. Leishman, Shai Barbut, Benjamin J. Wood, Christine F. Baes

**Affiliations:** 1 Department of Animal Biosciences, University of Guelph, Guelph, ON N1G 2W1 Canada; 2 Vereinigte Informationssysteme Tierhaltung W.V. (Vit), Heinrich-Schröder-Weg 1, 27283 Verden, Germany; 3 Department of Food Science, University of Guelph, Guelph, ON N1G 2W1 Canada; 4 Present Address: Hybrid Turkeys, 650 Riverbend Drive Suite C, Kitchener, ON N2K 3S2 Canada; 5 School of Veterinary Science, University of Queensland, Gatton, QLD 4343 Australia; 6 Institute of Genetics, Vetsuisse Faculty, University of Bern, 3001 Bern, Switzerland

**Keywords:** Functional analysis, GWAS, Myopathy, Pectoralis major, Turkey meat, White striping, Agricultural genetics, Animal breeding, Genomics

## Abstract

White striping (WS) is a myopathy of growing concern to the turkey industry. It is rising in prevalence and has negative consequences for consumer acceptance and the functional properties of turkey meat. The objective of this study was to conduct a genome-wide association study (GWAS) and functional analysis on WS severity. Phenotypic data consisted of white striping scored on turkey breast fillets (N = 8422) by trained observers on a 0–3 scale (none to severe). Of the phenotyped birds, 4667 genotypic records were available using a proprietary 65 K single nucleotide polymorphism (SNP) chip. The SNP effects were estimated using a linear mixed model with a 30-SNP sliding window approach used to express the percentage genetic variance explained. Positional candidate genes were those located within 50 kb of the top 1% of SNP windows explaining the most genetic variance. Of the 95 positional candidate genes, seven were further classified as functional candidate genes because of their association with both a significant gene ontology and molecular function term. The results of the GWAS emphasize the polygenic nature of the trait with no specific genomic region contributing a large portion to the overall genetic variance. Significant pathways relating to growth, muscle development, collagen formation, circulatory system development, cell response to stimulus, and cytokine production were identified. These results help to support published biological associations between WS and hypoxia and oxidative stress and provide information that may be useful for future-omics studies in understanding the biological associations with WS development in turkeys.

## Introduction

Great improvements have been made in poultry growth, efficiency, and meat yield^1^. Strategic improvements in management, nutrition, and genetic selection, have led to turkey toms that can weigh over 20 kg at 20 weeks of age^2^. However, some negative consequences of this improvement in growth and production are becoming apparent. White striping (WS) is a growth-related myopathy that is of increasing interest to the poultry industry. This myopathy presents itself as varying degrees of white striations on the surface of the muscle running parallel to the muscle fibers^3^. White striping is highly prevalent and has recently been shown to be as high as 88% (including mild to severe cases) in a population of purebred turkeys and as much as 60% in other turkey populations^4,5^. This myopathy has known negative consequences for consumer acceptance, nutritional, and functional quality of the product^3,6–9^. These aspects make research into the biological mechanisms behind the condition and potential methods of prevention of importance to the turkey industry.

Although the amount of research conducted on WS in turkeys is limited, there is more known about the myopathy in broiler chickens. Several studies have been conducted in broilers that investigated WS microscopically and showed that affected breasts have necrotic muscle tissue and increased presence of inflammatory cells, connective tissue, and fat^10–12^. While the exact mechanism for development of WS is still unknown, one of the main mechanisms proposed is ischemia in the affected muscle^13^. With the magnitude and speed of growth in modern genotypes, the limits of supporting physiological systems such as the circulatory and cardiovascular might have been reached. A major consequence of selection for muscle growth is an increase in muscle fiber hypertrophy^14–16^. This hypertrophy can then lead to insufficient vascularization and reduced blood supply to the fast-growing muscles^17–19^. A restriction in the circulatory system can lead to changes in stem cell growth and the accumulation of metabolic byproducts, inducing oxidative stress likely leading to necrosis, and increases in hypoxic conditions potentially impairing muscle cell regeneration, ultimately leading to the development of WS.

This mechanism of WS development has been supported through various -omics studies in broiler chickens at the level of the transcriptome^20–22^, proteome^23^, and metabolome^13^. However, there is a lack of research in these areas for turkeys. Consequently, the objective of this study was to investigate the genomic architecture of WS in turkeys through the estimation of genomic heritability and execution of a genome-wide association study (GWAS) followed by functional analysis for detection of metabolic pathways and gene ontologies associated with the myopathy.

## Results and discussion

### Estimation of genetic parameters

Genetic and phenotypic correlations of WS with other economically important traits were published in Vanderhout et al.^24^. Heritability of WS was estimated to be 0.20 ± 0.022 and is the first published genomic heritability estimate for WS in turkeys. The addition of genomic data resulted in a 33% increase in estimated heritability compared to pedigree information alone^24^. The present estimate was found to be within the range of previously published estimates of heritability (observed scale) in broiler chickens of 0.18–0.50^25–27^. The moderate heritability estimated in the present study suggests that there is a presence of genetic factors influencing WS that could potentially be exploited in selecting birds for reduced WS severity, however, environmental factors can also influence most of the phenotypic variance observed in the population^25^. However, it is worth noting that the comparing the present heritability estimate with what is reported in the literature may be challenging due to the different species being studied (chickens vs. turkeys), different methods of scoring WS (e.g., different levels of scoring), different breeding goals, and different prevalences of WS in the given populations. Differences in trait prevalence are well known to influence heritability estimates when using linear models which estimate parameters on the observed scale. The prevalence of WS in the present study ranged from 84 to 92% which is substantially greater than what was observed for Bailey et al.^25^ (18.5–33.8%), Lake et al.^27^ (79%), and Alnahhas et al.^26^ (50%). Therefore, it is reasonable to expect variability among studies with regard to heritability estimates.

### Significant SNP and positional candidate genes

**Figure 1:**
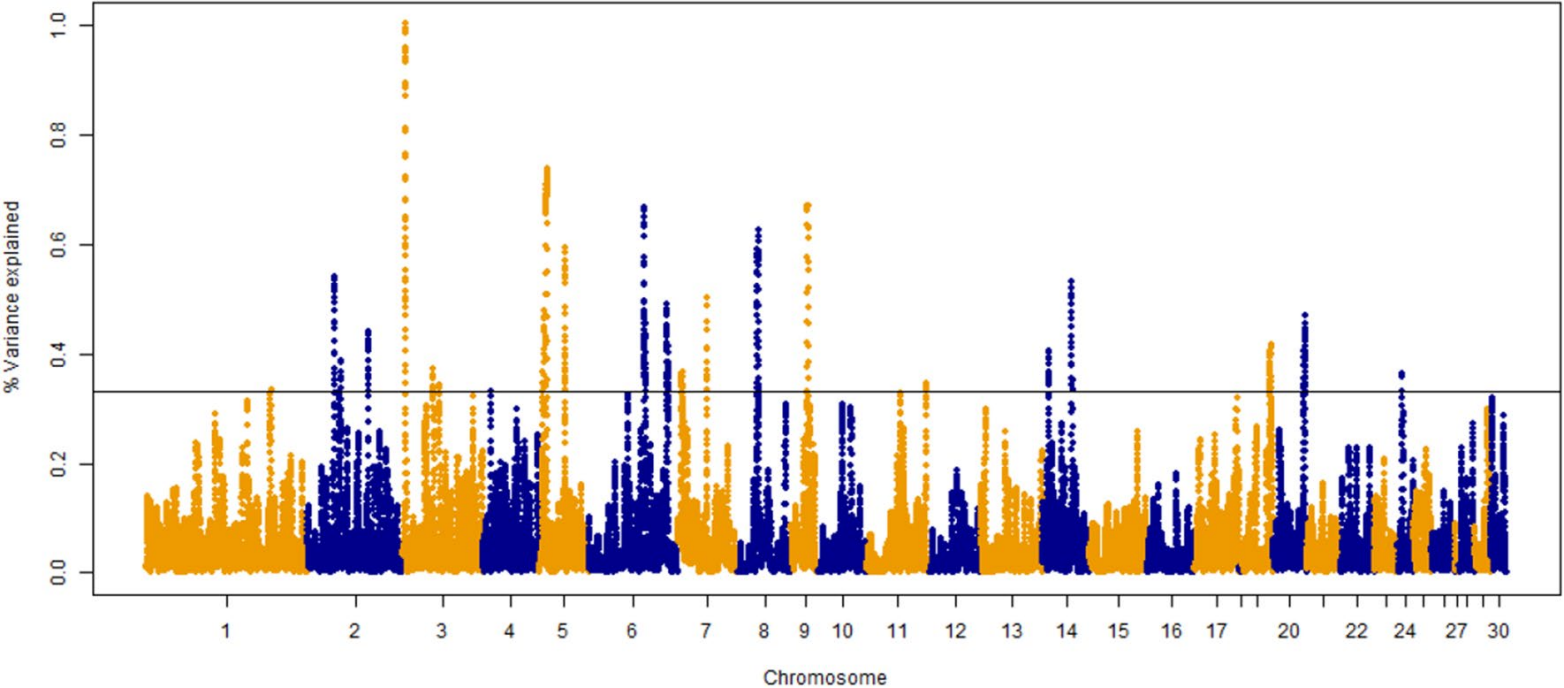
Manhattan plot for percentage of genetic variance explained by a 30-SNP sliding window across the genome for white striping severity score (0–3). The top 1% of SNP windows that explain the most genetic variance are located above the horizontal line (% of variance explained > 0.330%).

The percentage of genetic variance explained by each 30-SNP sliding window is presented in Fig. 1. Each window explained 0.05% of the genetic variance on average with no more than 1.00% of the variance being explained by any given window. This suggests that the inheritance of the trait is largely polygenic in nature. A total of 544 SNP windows were classified as significant (top 1% of variance explained) resulting in 95 positional candidate genes found within 50 kb upstream or downstream of these SNP. This distance has been suggested by Do et al.^28^ to be used when dealing with lower quality assemblies like that of the turkey. The positional candidate genes were located on *Meleagris gallopavo* autosomal chromosomes (MGA) 2 to 9, 11, 14, 19, 20, and 24. The 95 positional candidate genes were significantly associated (*p* < 0.05) with four KEGG metabolic pathways (Table 1) and 31 GO terms (21 BP, 3 CC, and 7 MF; Table 2). Positional candidate genes were further considered functional candidate genes (FCG) if they were associated with both a significant metabolic pathway and a significant GO term. Seven FCG were found and were involved mainly in the Wnt signaling pathway (*NFATc1*), RNA degradation (*LSM6* and *DHX36*), and focal adhesion (*COL6A3*, *FN1*, *VCL*, and *GRB2*).

**Table 1:** List of the KEGG metabolic pathways (p < 0.05) associated with the 95 positional candidate genes for white striping severity score (0–3) in turkeys.

Molecular pathway description	*p*-value	Gene names
One carbon pool by folate	< 0.01	*MTR*; *ATIC*
Wnt signaling pathway	0.01	NFATC1*; *SFRP4*; *CAMK2G*; *PPP3CB*
RNA degradation	0.01	LSM6*; DHX36*; *DCP1A*
Focal adhesion	0.05	COL6A3*; FN1*; VCL*; GRB2*

*Denotes functional candidate genes (genes associated with both a significant KEGG metabolic pathway and significant GO term).

**Table 2:** List of gene ontology terms including biological processes, cellular components, and molecular functions (p < 0.05) associated with the 95 positional candidate genes for white striping severity score (0–3) in turkeys.

GO ID	GO term	*p*-value	Gene names
Biological processes			
GO:0030031	Cell projection assembly	< 0.01	*ACTN2*; *TMEM216*; *TMEM138*; *NME8*; *RAB17*; VCL*; *ARHGEF26*
GO:0070925	Organelle assembly	< 0.01	*ACTN2*; *CSRP3*; *TMEM216*; *TMEM138*; *MLH1*; *NME8*; *RAB17*; *MYOZ1*; *BMP10*
GO:0003012	Muscle system process	< 0.01	*ACTN2*; *CSRP3*; *STAC*; *MYOZ1*; *BMP10*
GO:0060537	Muscle tissue development	< 0.01	*ACTN2*; *SMAD1*; *CSRP3*; *MYOZ1*; *BMP10*
GO:0030029	Actin filament-based process	< 0.01	*ACTN2*; *CSRP3*; *ELMO1*; *MYOZ1*; *MAPKAP1*; GRB2*; *BMP10*
GO:0043900	Regulation of multi-organism process	0.01	*CTDP1*; *TKFC*; *DDB1*; *GPR149*
GO:0009628	Response to abiotic stimulus	0.01	*CSRP3*; *DDB1*; *ARPP21*; *STAC*; DHX36*; *HSPA5*; GRB2*
GO:0034394	Protein localization to cell surface	0.02	*ACTN2*; VCL*
GO:0120036	Plasma membrane bounded cell projection organization	0.02	*ACTN2*; *TMEM216*; *TMEM138*; *NME8*; *RAB17*; FN1*; VCL*; *ZSWIM8*; *ARHGEF26*
GO:0032989	Cellular component morphogenesis	0.02	*ACTN2*; *CSRP3*; FN1*; VCL*; *ZSWIM8*; *MYOZ1*; *ARHGEF26*; *BMP10*
GO:0001816	Cytokine production	0.02	NFATC1*; *CD6*; *TKFC*; FN1*; DHX36*
GO:0040007	Growth	0.02	*ADNP2*; *SMAD1*; FN1*; VCL*; *MYOZ1*; *GPR149*; *BMP10*
GO:0097435	Supramolecular fiber organization	0.02	*ACTN2*; *TPPP*; *CSRP3*; *MYOZ1*; GRB2*; *BMP10*
GO:0007264	Small GTPase mediated signal transduction	0.03	*ELMO1*; *RAB17*; *ARHGEF26*; *MAPKAP1*; GRB2*
GO:0061061	Muscle structure development	0.03	*ACTN2*; NFATC1*; *CSRP3*; *MYOZ1*; *BMP10*
GO:0051640	Organelle localization	0.04	*ABCE1*; *MLH1*; *STARD3NL*; *RAB17*; *AP3M1*
GO:0072359	Circulatory system development	0.04	*ACTN2*; NFATC1*; *SMAD1*; *CSRP3*; FN1*; DHX36*; *BMP10*
GO:0048646	Anatomical structure formation involved in morphogenesis	0.04	*ACTN2*; *SMAD1*; *CSRP3*; FN1*; *MYOZ1*; GRB2*; *BMP10*
GO:0034330	Cell junction organization	0.04	*ACTN2*; FN1*; VCL*
GO:0000003	Reproduction	0.05	*ARID4B*; *TRIP13*; *SMAD1*; *MLH1*; *GPR149*; DHX36*
GO:0104004	Cellular response to environmental stimulus	0.05	*DDB1*; DHX36*; GRB2*
Cellular components			
GO:0042383	Sarcolemma	< 0.01	*STAC*; COL6A3*; VCL*
GO:0099080	Supramolecular complex	< 0.01	*ACTN2*; *TPPP*; *CSRP3*; FN1*; VCL*; *MYOZ1*; *BMP10*
GO:0120114	Sm-like protein family complex	0.03	*TXNL4A*; LSM6*
Molecular functions			
GO:0051020	GTPase binding	0.02	*ELMO1*; *AP3M1*; *ARHGEF26*; *GAPVD1*; *MAPKAP1*
GO:0008092	Cytoskeletal protein binding	0.02	*ACTN2*; *TPPP*; *CSRP3*; *NME8*; VCL*; *MYOZ1*; *BMP10*
GO:0044877	Protein-containing complex binding	0.02	*ACTN2*; *CTDP1*; *ABCE1*; *DDB1*; *MLH1*; FN1*; VCL*
GO:0019904	Protein domain specific binding	0.03	*ACTN2*; *DDB1*; *ELMO1*; FN1*; GRB2*
GO:0044325	Ion channel binding	0.04	*ACTN2*; *STAC*
GO:0003697	Single-stranded DNA binding	0.05	*MLH1*; DHX36*
GO:0060090	Molecular adaptor activity	0.05	*DDB1*; GRB2*

*Denotes functional candidate genes (genes associated with both a significant KEGG metabolic pathway and significant GO term).

Due to WS being a growth-related myopathy, it is not surprising that several growth and muscle development related BP terms (GO:0003012 muscle system process, GO:0040007 growth, and GO:0061061 muscle structure development) were found to be significantly overrepresented (*p* < 0.05) by the 95 positional candidate genes. The large selection pressure placed on growth for economically significant muscle groups (i.e., *pectoralis*) has resulted in meat producing birds that are likely reaching the limit of supporting systems, such as the circulatory system. Thus, the proposed mechanism of WS development is primarily thought to be related to poor blood flow in the breast muscle leading to hypoxia, pressure on satellite cells, and oxidative stress^13,21,29^. The highly conserved Wnt signalling pathway, one of the four significant metabolic pathways, plays an important role in both embryonic development, where it regulates processes such as differentiation and cell proliferation, polarity, and migration, as well as post-natally, where it regulates tissue homeostasis and biological processes involved in many disorders and cancers^30–32^. The FCG associated with the Wnt signalling pathway, nuclear factor of activated T cells 1 (*NFATc1*), is found on MGA3 associated with the largest peak in variance explained. This gene has been shown to play a large role in cell cycle progression of human aortic smooth muscle cells^33^ and promoting the response to injury in arterial smooth muscle cells^34^. The effect of *NFATc1* and the Wnt signalling pathway in the development and repair of the vascular system may be what leads to its significant relationship to WS. Some significant BP terms found in the present study were associated with FCG (including *NFATc1*, *DHX36*, and *FN1*), specifically GO:0009628 response to abiotic stimulus (*p* = 0.01) and GO:0072359 circulatory system development (*p* = 0.04), further supporting the relationship between hypoxia, oxidative stress, and WS.

Functional candidate genes collagen type VI alpha 3-chain (*COL6A3*) and fibronectin 1 (*FN1*) were also previously found to be significantly associated with WS in broiler chickens^35^ and differentially expressed between broiler chicken breasts affected versus not affected by WS^36,37^. The *COL6A3* gene produces collagen found in the extracellular matrix of cells that make up skeletal muscles, and mutations in the gene are associated with muscle weakness, atrophy, and necrosis in humans^38^. The *FN1* gene encodes a glycoprotein which plays a role in the creation of extracellular matrix structures during tissue repair and increases in the expression of this gene have been linked with Duchenne muscular dystrophy in humans^39^. Given the increase in fat and connective tissue that replaces damaged muscle tissue in affected breast muscles, the link between these two genes and WS is reasonable. Another gene of interest is cytosine and glycine rich protein 3 (*CSRP3*), a positional candidate gene found to be associated with several significant GO terms including 10 BP, one CC, and 1 MF. The *CSRP3* gene has been previously shown to be upregulated in broiler chicken breasts affected with WS^22,40^. This gene encodes a muscle LIM protein and overexpression of such protein can promote muscle differentiation, regeneration, and structural repair of skeletal muscle^41,42^ further emphasizing the link between WS and muscle tissue damage.

The BP term, GO: 0001816 cytokine production, was found to be significantly overrepresented (*p* = 0.02) by the positional candidate genes in the present study, including three of the seven FCG (*NFATc1*, *FN1*, and *DXH36*). A microscopic characteristic consistently found in poultry breast tissue affected by WS is an elevated presence of inflammatory cells and cytokines^10,43,44^. Cytokines are small proteins that play a large role in immune response and inflammation and the elevated presence of these molecules in the muscle of affected breasts is symbolic of muscle cell injury^45,46^. Whether these genes, and subsequent production of cytokines, was upregulated in the affected breasts of the current study is unknown, however, the expression of inflammatory cytokine genes has been shown to increase with increasing severity of WS in broiler chickens^44^.

To the best of our knowledge, this study provides the first published estimate of genomic heritability of WS in turkeys and provides the first look into the genomic architecture of WS in turkeys by means of a GWAS and functional analysis. The heritability estimate of WS was found to be 0.20 ± 0.022, and results of the GWAS emphasize the polygenic nature of the trait with no specific genomic region contributing a large portion to the overall genetic variance. Results of the functional analysis identified four significant KEGG metabolic pathways, 31 significant GO terms (21 BP, 3 CC, and 7 MF) and seven functional candidate genes associated with WS. Overall, pathways relating to growth, muscle development, collagen formation, circulatory system development, cell response to stimulus, and cytokine production were highlighted. The results of the present study provide support for the oxidative stress and hypoxic theory of WS development. It should be noted that the WS phenotype was analyzed using a linear model which may reduce the statistical power when considering categorical traits (i.e., compared to a threshold model). Continued -omics research on the topic of WS in turkeys is recommended to further identify relationships between the myopathy and biological processes to identify improved prevention methods. For example, using a meta-GWAS approach to provide a comprehensive assessment of genetic factors influencing WS. Future research should also focus on developing methods of quantitatively scoring WS using technologies such as machine vision algorithms. Such measures would permit an increase in phenotypic measures increasing the power of future analyses.

## Materials and methods

### Animals

All protocols complied with the guidelines of the Canadian Council on Animal Care and were approved by the University of Guelph Animal Care Committee (AUP 3782). The study was conducted in accordance with relevant guidelines and regulations as well as the ARRIVE guidelines^47^. Adult male turkeys (20–24 weeks old) from three purebred genetic lines (A, B, and C) were processed over 44 weeks between July 2018 and November 2019. The genetic lines included a sire-line with selection focused on body weight, meat yield, and feed efficiency (line A), a dam-line that was selected primarily for body weight and reproductive traits (line B), and a dam-line selected mainly for reproductive traits (line C). Birds were reared under identical housing and management conditions as specified by the breeding company management guidelines (Hybrid Turkeys, 2020). During processing at a commercial poultry processing plant, birds were electrically stunned, exsanguinated, scalded, defeathered, and eviscerated before moving to the water chiller. Upon completion of the 24 h chilling period (40 min in 5 °C water, 1.5–2 h in 1–2 °C water, and remainder of time layered in ice), birds were deboned, and meat quality and breast muscle weights were measured.

### Phenotype and genotype data

Summary statistics of the data are shown in Table 3. Deboned *Pectoralis major* muscles (N = 8422) were photographed (Hero 6, GoPro, San Mateo, CA, USA) approximately 24 h post-mortem. Photographs were taken using the *normal* focal length setting from approximately 40 cm above the surface of the breast. The photographs were randomly assigned to six observers who scored the breasts for WS using a 0–3 scoring scale adapted from a system developed in broiler chickens after testing the reliability of the system^5,7^. In brief, a score of 0 indicated no or minimal white striations whereas a score of 3 indicated the presence of thick white striations covering the breast. Genotypes were collected on 4667 birds using a proprietary 65 K single nucleotide polymorphism (SNP) array (65,000 SNP; Illumina, Inc.). PLINK software^48^ was used for quality control and SNP markers located on non-autosomal regions with minor allele frequency lower than 0.05, call rate lower than 90%, or significantly deviating from Hardy Weinberg proportions (*p* < 1 × 10^–8^) were removed. The quality control resulted in 54,407 markers retained for analysis.

**Table 3:** Summary statistics of each genetic line (A, B, C) of turkeys.

	Line A	Line B	Line C
Number of phenotypic records	2856	1838	3728
White striping score 0 (frequency)	450 (0.16)	155 (0.08)	362 (0.10)
White striping score 1 (frequency)	1181 (0.41)	565 (0.31)	1137 (0.30)
White striping score 2 (frequency)	1128 (0.40)	950 (0.52)	1809 (0.49)
White striping score 3 (frequency)	97 (0.03)	168 (0.09)	420 (0.11)
Mean slaughter age in days (SD)	144.13 (3.95)	149.68 (2.10)	153.65 (3.63)
Number of animals in the pedigree	6530	4146	6063
Number of genotyped birds (with phenotypic data)	963 (766)	477 (431)	1,185 (1059)
Number of genotyped sires in pedigree	126	153	183
Number of genotyped dams in pedigree	623	354	583
Total number of SNP markers (all lines)	54,407		

### Statistical analysis

A linear mixed model was used to estimate variance components through restricted maximum likelihood using the BLUPf90 family of programs^49^. The linear mixed model used can be described as follows:

$${\mathbf{y}} = {\mathbf{Xb}} + {\mathbf{Za}} + {\mathbf{e}},$$

where **y** is the vector of WS scores; **b** is a vector of fixed effects including genetic line (3 levels: A, B, and C), hatch week-year (58 levels), age at slaughter (7 levels; 141–163 days), and score observer (6 levels); **a** is a vector of additive genetic effects distributed as $\mathbf{a}\sim N(0, \mathbf{H}{\sigma }_{a}^{2})$, where **H** is the combined pedigree-genomic relationship matrix as in Aguilar et al. (2010) constructed using the PREGSf90 program^49^. ${\sigma }_{a}^{2}$ is the additive genetic variance; **e** is the vector of residual effects which has a distribution of $\mathbf{e}\sim N(0,{\sigma }_{e}^{2})$ where ${\sigma }_{e}^{2}$ is the residual variance; and **X** and **Z** are design matrices relating the observations to the fixed and random effects, respectively.

Estimates of SNP effects were derived from the estimated genomic breeding values (**gEBV**) following^50^, using a weighted genomic relationship matrix:

$$\widehat{\mathbf{g}}=\mathbf{D}{\mathbf{Z}}^{\mathrm{^{\prime}}}{[\mathbf{Z}\mathbf{D}{\mathbf{Z}}^{\mathrm{^{\prime}}}]}^{-1}{\widehat{\mathbf{u}}}_{{\text{g}}},$$

where $\widehat{\mathbf{g}}$ is a vector of SNP marker effects; **D** is a diagonal matrix of weights for variances of SNPs; **Z** is a matrix relating genotype of each locus; and ${\widehat{\mathbf{u}}}_{{\text{g}}}$ is the vector of gEBV. Due to the proposed polygenic nature of WS and the relatively poor annotation of the turkey genome, a 30-SNP sliding window approach was utilized. This approach allows for accumulating the variance explained by each set of 30 adjacent SNP, which would lead to identify potential genomic regions associated with WS that may not be detected due to the low variance explained by single SNPs. These analyses were carried out using the BLUPf90 family of programs^49^.

### Functional analysis

An arbitrary threshold for markers in the 99th percentile of variance explained were considered significant. Using the Turkey 5.1 assembly^51^, positional candidate genes within ± 50 kb of the significant SNP were retrieved using the Ensembl Genes database version 104 (https://useast.ensembl.org/Meleagris_gallopavo/Info/Index) implemented through the GALLO R package^52^. Gene ontology (GO) enrichment analysis including biological processes (BP), cellular components (CC), and molecular functions (MF) as well as metabolic pathway analysis using the Kyoto Encyclopedia of Genes and Genomes (KEGG) database^53^ were performed on the positional candidate genes using the WebGestaltR R package^54^ and the *Gallus gallus* database.

## Data Availability

The datasets generated during and/or analysed during the current study are available from the corresponding author on reasonable request.
